# Whole-Genome Sequencing of Streptomycin-Resistant *Mycobacterium tuberculosis* Isolate VRFCWCF MRTB 180 Reveals Novel and Potential Mutations for Resistance

**DOI:** 10.1128/genomeA.00919-14

**Published:** 2014-09-11

**Authors:** Gayathri Ramasubban, Dhanurekha Lakshmipathy, Umashankar Vetrivel, Lily Therese Kulandai, Hajib Narahari Madhavan, R. Sridhar, N. Meenakshi

**Affiliations:** a L&T Microbiology Research Centre, Vision Research Foundation, Chennai, India; b Centre for Bioinformatics, Vision Research Foundation, Chennai, India; c Department of Thoracic Medicine, Stanley Medical College, Chennai, India; d Institute of Thoracic Medicine, Chetpet, Chennai, India

## Abstract

We announce the draft genome sequence of a streptomycin monoresistant *Mycobacterium tuberculosis* strain (VRFCWCF MRTB 180) isolated from sputum of a clinically suspected tuberculosis patient.

## GENOME ANNOUNCEMENT

Streptomycin (STR), an aminocyclitol glycoside, is an alternative first line anti-tuberculosis (TB) drug recommended by the WHO (1). STR is therefore used in the re-treatment of TB cases together with the four-drug regimen that includes isoniazid, rifampin, pyrazinamide, and ethambutol (2). The effect of STR has been demonstrated to take place at the ribosomal level. STR interacts with the 16S rRNA and S12 ribosomal protein (*rrs* and *rpsL*) (3, 4), inducing ribosomal changes, which cause misreading of the mRNA and inhibition of protein synthesis.

We announce the draft genome sequence of a STR resistant *M. tuberculosis* (VRFCWCF MRTB 180) strain, isolated from sputum of a clinically suspected tuberculosis patient. Whole-genome sequencing was performed using an Ion Torrent PGM platform as mentioned in our previous works (5, 6). The generated sequence reads were ﬁltered with a Phred score cutoff of ≥20. The ﬁltered sequences were *de novo* assembled using MIRA Assembler 3.4.1.1, wherein 119 contigs totaling 4,327,456 bp in length, with 134.19× coverage and an *N*_50_ length of 78,746 bp, were obtained. These sequences were further ordered and reoriented with *M. tuberculosis* H37Rv (accession no. NC_000962.3) as a reference using Mauve (7) and in-house written scripts. Furthermore, the assembled sequences were also subjected to annotation by NCBI PGAAP (http://www.ncbi.nlm.nih.gov/genomes/static/Pipeline.html), which revealed 3,825 protein-coding genes and 55 RNA-coding genes.

In addition, BLAST and multalign analysis revealed the presence of 4 novel non-synonymous substitution mutations coding for streptomycin resistance. The nucleotides at positions 815,236 (TCG → CCG), 1,853,974 (CAC → TAC), 3,861,914 (GCC → TCC), and 3,876,953 (CGA → CAA) lead to amino acid variations Ser to Pro in the *rplO* region, His to Tyr in the *SpoU* region, Ala to Val in the *rpsI* region, and Arg to Gln in the *rplQ* region, respectively. These mutations were carefully inspected through the genome alignment viewer of CLC genomics Workbench 6.5. As these mutations are in the protein coding regions, further structural bioinformatics of these proteins will lead to a better understanding of the molecular basis of streptomycin resistance. This is the first study on whole-genome sequencing of a streptomycin monoresistant strain from India.

### Nucleotide sequence accession numbers.

This whole-genome shotgun project has been deposited at DDBJ/EMBL/GenBank under the accession no. JMJH00000000. The version described in this paper is version JMJH00000000.1.
